# Marker discovery and associations with β-carotene content in Indian dairy cattle and buffalo breeds

**DOI:** 10.3168/jds.2019-16361

**Published:** 2019-08-30

**Authors:** F. Bertolini, J. Chinchilla-Vargas, J. R. Khadse, A. Juneja, D. Deshpande, K. Bhave, V. Potdar, P. M. Kakramkar, A. R. Karlekar, A. B. Pande, Rohan L. Fernando, M. F. Rothschild

**Affiliations:** 1 Department of Animal Science, Iowa State University, 2255 Kildee Hall, 806 Stange Road, Ames 50011; 2 National Institute of Aquatic Resources, Technical University of Denmark, Kemitoryet 2800, KGs. Lyngby, Denmark; 3 Bharatiya Agro Industries Foundation, Development Research Foundation, Bhavan, Dr. Manibhai Desai Nagar Warje, Pune 411058, India

**Keywords:** beta-carotene, single nucleotide polymorphism, milk, cattle, buffalo

## Abstract

Vitamin A is essential for human health, but current intake levels in many developing countries such as India are too low due to malnutrition. According to the World Health Organization, an estimated 250 million preschool children are vitamin A deficient globally. This number excludes pregnant women and nursing mothers, who are particularly vulnerable. Efforts to improve access to vitamin A are key because supplementation can reduce mortality rates in young children in developing countries by around 23%. Three key genes, *BCMO1, BCO2*, and *SCARB1*, have been shown to be associated with the amount of β-carotene (BC) in milk. Whole-genome sequencing reads from the coordinates of these 3 genes in 202 non-Indian cattle (141 *Bos taurus*, 61 *Bos indicus*) and 35 non-Indian buffalo (*Bubalus bubalis*) animals from several breeds were collected from data repositories. The number of SNP detected in the coding regions of these 3 genes ranged from 16 to 26 in the 3 species, with 5 overlapping SNP between *B. taurus* and *B. indicus*. All these SNP together with 2 SNP in the upstream part of the gene but already present in dbSNP (https://www.ncbi.nlm.nih.gov/projects/SNP/) were used to build a custom Sequenom array. Blood for DNA and milk samples for BC were obtained from 2,291 Indian cows of 5 different breeds (Gir, Holstein cross, Jersey Cross, Tharparkar, and Sahiwal) and 2,242 Indian buffaloes (Jafarabadi, Murrah, Pandharpuri, and Surti breeds). The DNA was extracted and genotyped with the Sequenom array. For each individual breed and the combined breeds, SNP with an association that had a *P*-value <0.3 in the first round of linear analysis were included in a second step of regression analyses to determine allele substitution effects to increase the content of BC in milk. Additionally, an *F*-test for all SNP within gene was performed with the objective of determining if overall the gene had a significant effect on the content of BC in milk. The analyses were repeated using a Bayesian approach to compare and validate the previous frequentist results. Multiple significant SNP were found using both methodologies with allele substitution effects ranging from 6.21 (3.13) to 9.10 (5.43) μg of BC per 100 mL of milk. Total gene effects exceeded the mean BC value for all breeds with both analysis approaches. The custom panel designed for genes related to BC production demonstrated applicability in genotyping of cattle and buffalo in India and may be used for cattle or buffalo from other developing countries. Moreover, the recommendation of selection for significant specific alleles of some gene markers provides a route to effectively increase the BC content in milk in the Indian cattle and buffalo populations.

## INTRODUCTION

Vitamin A plays a key role in human health. Inclusion of proper amounts of vitamin A in the diet is a key factor for the development and maintenance of healthy vision (Bennasir et al., 2010), proper functioning of the immune system (Hussey and Klein, 1990), improved red blood cell and hemoglobin production (Lynch, 1997), in addition to prevention of diseases such as Alzheimer’s and schizophrenia (Davis et al., 1991; Goodman and Pardee, 2003). Moreover, vitamin A is related to successful growth in early childhood and embryonic development (Semba, 1998). According to the World Health Organization, 250 million children are vitamin A deficient worldwide and improving access to vitamin A can have a big effect, especially in developing countries such as India.

Vitamin A supplementation through naturally or artificially fortified food can reduce mortality rates in young children by about 23% (Beaton et al., 1993). Beta-carotene (**BC**) could also replace vitamin A, as it can be metabolized to vitamin A after ingestion (Bennasir et al., 2010). Beta-carotene is fat soluble, and thus it is most efficiently absorbed in the presence of fat components. Therefore, milk is an ideal food for its delivery (Ribaya-Mercado, 2002). Consequently, selection for increased BC content in milk could be a good approach to improve the nutritional value of milk (Berry et al., 2009).

Genomic technologies have recently facilitated the identification of 3 key genes in BC metabolism: β-carotene oxygenase 1 (*BCMO1* or *BCO1*) and β-carotene oxygenase 2 (*BCMO2* or *BCO2*), which are involved in the cleavage of β-carotene (D’Ambrosio et al., 2011), and scavenger receptor class B member 1 (*SCARB1*), which is involved in cellular transport (Valacchi et al., 2011). A QTL related to milk BC content linked to the *BCO2* gene has been officially reported and subsequent research revealed allelic variants that are associated with different amounts of BC in milk (Berry et al., 2009). These findings have suggested that selection for beneficial alleles could improve BC levels in milk. The aim of this work was to map and identify SNP in the 3 candidate genes *BCMO1, SCARB1*, and *BCO2* in cattle and buffalo with next-generation sequencing resources available, develop a SNP panel, and use this panel to detect SNP that are associated with BC content in several Indian cattle and buffalo breeds.

## MATERIALS AND METHODS

### Animal Care

The sample collection done for the purpose of this project was performed using animal care procedures approved by Iowa State University (Institutional Animal Care and Use Committee Log # 7–15–8061-B) and the Bharatiya Agro Industries Foundation veterinarian from the Bharatiya Agro Industries Foundation research foundation meeting the required standards in India and with approval from the Bill and Melinda Gates Foundation.

### High-Throughput SNP Discovery and Building of the Sequenom Custom Panel

Reads from whole-genome sequencing of 202 cattle (141 *Bos taurus*, 35 *Bos indicus*) and 61 buffalo (*Bubalus bubalis*) non-Indian animals from several breeds were collected from SRA (Sequence Nucleotide Archive; https://www.ncbi.nlm.nih.gov/sra) database (*B. taurus*) as part of the 1000 bulls genome project (Daetwyler et al., 2014), from the International Buffalo Consortium (Sonstegard et al., 2012; *B. bubalis*), or from other projects (e.g., Stafuzza et al., 2017, or data not shown; *B. indicus*). The list of breeds is reported in Supplemental Table S1 (https://doi.org/10.3168/jds.2019-16361). Because of the high genomic similarity between *B. taurus* and *B. indicus*, reads from both species were aligned against the same UMD3.1 (https://www.ncbi.nlm.nih.gov/assembly/GCF_000003055.6) reference genome (GCA_000003055.3). *Bubalus bubalis* reads were aligned to the most recent buffalo reference genome available at the time of the analysis: MD_CAS-PUR_WB_2.0 (GCA_000471725.1). Coordinates of the *BCMO1, BCO2*, and *SCARB1* genes were retrieved through Ensembl (www.ensembl.org) for *B. taurus* and through alignment (https://blast.ncbi.nlm.nih.gov/Blast.cgi) of the coding sequence of the 3 genes against the UMD3.1 and MD_CASPUR_WB_2.0 (https://www.ncbi.nlm.nih.gov/assembly/GCF_000471725.1) genomes for *B. indicus* and *B. bubalis*, respectively.

For *B. taurus*, bam files corresponding to the genomic coordinates of the 3 genes were retrieved directly from the SRA database. For *B. indicus* and *B. bubalis*, Burrows-Wheeler aligner (BWA MEM) with standard conditions (Li and Durbin, 2010) and Samtools (Li, 2011) were used to align the reads to the respective reference genomes and extract the portions corresponding the genomic coordinates of the 3 genes. To remove reads with ambiguous alignments to repetitive regions in the genome, we arbitrarily selected a minimum mapping quality score threshold of 10, which corresponds to a 10% chance of alternative alignment, to filter our reads (Hwang et al., 2019). After that, the standard pipeline of the Samtools (Li, 2011) or GATK (McKenna, 2017) software was applied to call the variants in all the samples for the *BCMO1, BCO2*, and *SCARB1* genes, where only SNP with SNP quality ≥30 and at least 6× as coverage depth in at least one animal (or at least 2 for *B. taurus*) were considered. For cattle, the effect of each SNP was evaluated through variant effect predictor (McLaren et al., 2016). For buffalo SNP, because of the lack of variant effect information, the effect was determined comparing the predicted protein sequence output derived by changing one allele at the time with the reference protein sequence using the BLAST web tool (https://blast.ncbi.nlm.nih.gov/Blast.cgi).

Comparing the AA changes, we were able to determine if a mutation in the coding region could be synonymous (when no AA change was detected) or nonsynonymous (when the mutation causes AA change in the protein frame comparing to the refseq protein).

The SNP located in the coding region or in untranslated region or already reported in dbSNP were considered for a Sequenom panel. For each selected SNP, probes for the panel were designed by Geneseek (Lincoln, NE). The panel (composed of 67 SNP as it will be shown in results) was then tested by genotyping a subset of Indian samples belonging to 5 different breeds (Supplemental Table S2, https://doi.org/10.3168/jds.2019-16361), where DNA was extracted from blood following standard protocols. Each animal was genotyped in triplicate.

### BC Measurement and SNP Genotyping

Beta-carotene concentration was measured from milk samples collected through HPLC for 2,291 cattle (Holstein cross, Jersey cross, Sahiwal, Tharparkar, and Gir) and 2,242 buffalo (Jaffrabadi, Murrah, Pandharpuri, Mehsana, and Surti), as shown in Supplemental Table S3 (https://doi.org/10.3168/jds.2019-16361). For each animal, information on lactation, milk yield, location, and farmer was also collected if available. The same animals were genotyped with the previously developed Sequenom custom array and for each breed, only SNP with call rate ≥0.90 and belonging to the same species (cattle and buffalo) were considered. For the combined analyses within species, SNP had to have a call rate >90% for all the breeds to be included. The retained missing SNP were then imputed breed by breed using Fimpute 2.2 (Sargolzaei et al., 2014). Pairwise linkage disequilibrium (**LD**) among SNP of the same genes were calculated with Haploview software (Barrett et al., 2005). Pairwise SNP with r^2^ > 0.6 were considered as in strong LD.

### Statistical Association Analyses

For each SNP, the genotypes were coded as 0 for homozygous for one of the alleles, 1 for heterozygous genotypes, and 2 for homozygous for the other allele. After this, ANOVA with proc GLM in SAS 9.4 (SAS Institute Inc., 2013) were performed initially using 3 different linear models for each breed of cattle and buffalo and for each species: (1) the first linear model included only non-SNP fixed effects, depending on the availability of information on location, farmer, and breed; (2) to examine the contribution of SNP to the variability of BC content in milk, all SNP were added to the first model as covariates; and (3) finally, only SNP that showed a *P*-value less than 0.3 were included in the model to estimate their additive effects, using the “solutions” option in SAS 9.4 (SAS Institute Inc., 2013). Further, when available, the number of lactations and milk yield were included in the models as covariates. Additionally, in the case of buffaloes, a fixed effect of batch was included in all the models. The reason for this being that data for buffaloes were collected at 2 separate periods of time with marked differences in precipitation that might have had an effect on the concentration of BC in milk. The option of correcting for multiple testing with adaptive false discovery rate (data not shown) was explored, and the number of significant SNP that dropped out was as expected. However, given the limited number of SNP tested and the previous SNP filtering steps taken, it was finally decided that correcting for multiple tests was not necessary and would reduce the information desired.

There is no known history of selection for BC content in the population studied. Because of this, the last step of this analysis was to contrast the hypothetical extreme cases of animals that were homozygous for all the favorable alleles (*P* < 0.3) with animals that were homozygous for the unfavorable alleles with the objective of demonstrating the effect that long-term sustained selection could have on the BC content in milk. These analyses were performed both for each individual gene and across all 3 genes for each species and the combined breeds as well.

Suppose SNP covariates are coded as 0, 1, or 2 (number of copies of the A allele). Then, at locus *j*, if the A allele is favorable, the substitution effect, *β_j_*, is positive, and if it is unfavorable, *β_j_* is negative. So, if locus *j* is favorable, the difference between the favorable and unfavorable homozygotes will be 2 × *β_j_*. On the other hand, if locus *j* is unfavorable, this difference becomes 2 × –*β_j_*. Thus, at an arbitrary locus, the difference between the favorable and unfavorable homozygotes is 2 × |*β_j_*|, and across all the loci, the difference between the most favorable and least favorable genotypic values is *D* = 2Σ*_j_*|*β_j_*|. But, as *β_j_* is not observed, *D* is estimated as D^=2Σj|β^j|. These analyses were performed using proc GLM in SAS 9.4 (SAS Institute Inc., 2013) along with the estimate function to obtain coefficients, standard errors, and a nominal *P*-value for the difference between the genotype contrasted.

In addition to the previously described frequentist approach, a Bayesian analysis was also undertaken with a model that considered the non-SNP effects as fixed and all SNP effects as random. It has been recognized that explicit adjustments for multiple tests are not needed when inference is based on posterior probabilities (Stephens and Balding, 2009; Gelman et al., 2012; Chen et al., 2017; Fernando et al., 2017), and thus, the Bayesian analysis does not suffer from the multiple-test penalty (Stephens and Balding, 2009). As adjustments for multiple testing were not undertaken in the frequentist approach, the Bayesian approach provides a useful validation of the results from the frequentist approach.

Initially a Bayes A prior was employed given that Bayes A assumes that all the markers included in the model have an effect on the phenotype. Thus, using only the markers that showed a nominal *P*-value <0.3 in the previous steps, it was assumed this would produce accurate predictions. Additionally, to examine the usefulness of another Bayesian prior for inference of the SNP effects, 2 sets of simulations were employed. The simulated data set was composed of 500 observations and 20 markers. In the first set, no markers had an effect on the phenotype, whereas on the second set 25% of the markers had an effect on the phenotype. These simulated data were tested with Bayes A (Meuwissen et al., 2001), where SNP substitution effects are a priori assumed to be identically and independently distributed *t* random variables and Bayes Cπ (Habier et al., 2011), where they are assumed to be identically and independently distributed with a point mass at 0 with probability π or normally distributed with probability 1 – π. Further, in Bayes Cπ, π is assumed unknown with a uniform prior between 0 and 1. For both simulated data sets, Bayes Cπ consistently produced more accurate estimates of the contrast between homozygotes for all favorable versus all unfavorable alleles than Bayes A (simulated data results not shown).

With this evidence, an additional Bayesian analysis was undertaken using the Bayes Cπ prior for SNP effects. Inferences on marker effects were then based on their posterior distributions, which were estimated from Markov chain Monte Carlo samples obtained using the JWAS (Cheng et al., 2018) package.

## RESULTS AND DISCUSSION

### SNP Discovery and Panel Performance

Milk is a rich source of BC, which is one of several naturally occurring carotenoids, and BC is also abundantly available in plants (fruits and vegetables) that humans obtain through foods of plant origin (Olson, 1999). Beta-carotene is present in cow milk and at lower levels in buffalo (Ullah et al., 2017). In this study, an initial breed and species comparison demonstrated differences in BC level in milk among cattle and buffalo but shows that BC can be detected also in buffalo breeds, with Jafarabadi having a concentration higher than most of the cattle breeds considered (Table 2). Beta-carotene can be acquired from the bloodstream by various tissues within the body, to be stored or be readily metabolized (Shete et al., 2013). Apart from its excretion in milk, BC also accumulates in the liver and other tissues (Schmitz et al., 1991). Three key genes have been considered for our analyses, as they were previously reported to be associated with BC levels and are directly involved in BC metabolism. The first is the *BCMO1* gene, which symmetrically cleaves BC into 2 molecules of retinal using a dioxygenase mechanism. The role of *BCMO1* in BC conversion efficiency has been clarified, as well as a report of a genetic variation in humans that can affect BC conversion efficiency (Lindqvist et al., 2007). The *BCO2* gene is also involved in the cleavage of BC, through asymmetrical cleavage (Amengual et al., 2011). A SNP corresponding a stop codon in this gene has been associated with BC content in milk in cattle (Berry et al., 2009). The third gene, *SCARB1*, is involved in cellular uptake of several provitamin A carotenoids, including BC. Genetic variation associated with plasma BC was also reported (Borel et al., 2013), as well as a variation related to carotenoid-based coloration in birds (Toomey et al., 2017).

**Figure 1:**
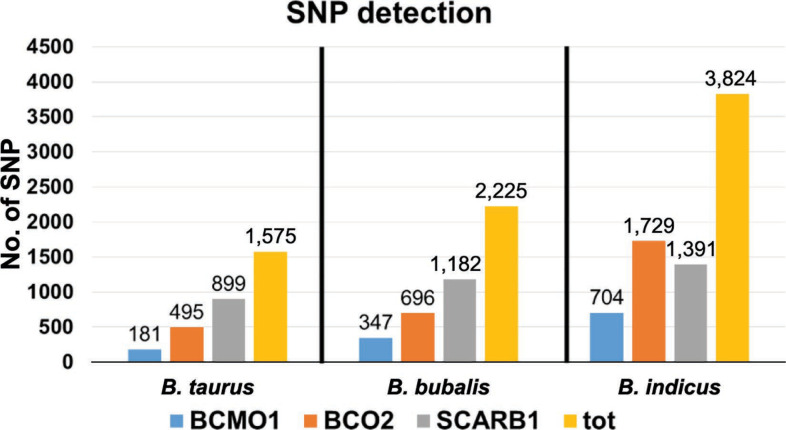
Numbers of SNP and their distribution derived by next-generation sequencing data across the 3 species (*Bos taurus, Bubalus bubalis*, and *Bos indicus*). tot = total.

Overall a total of 1,576 SNP were detected across the 3 genes for *B. taurus*, 2,225 for *B. indicus*, and 3,824 for *B. bubalis* (Figure 1). The differences in number of SNP are probably due to the crossbreds considered and the differences between the 2 species. Only 2 breeds for *B. taurus* (Holstein and Jersey) were considered for the analyses because these 2 are extensively employed around the world and are often crossbreed with *B. indicus* (FAO, 2013). A total of 67 SNP met the defined parameters [(a) in a coding region, (b) in the gene untranslated region, or (c) reported in dbSNP], and thus, were selected to compose the SNP panel (Table 1) and were divided as follows: a total of 17 SNP targeted for *B. taurus* (6 SNP for *BCMO1*, 5 for *BCO2*, and 6 for *SCARB1*), 27 SNP for *B. indicus* (10 SNP for *BCMO1*, 7 for *BCO2*, and 10 SNP for *SCARB1*) and 23 SNP for *B. bubalis* (8 for *BCMO1*, 6 for *BCO2*, and 9 for *SCARB1*). For convenience, they were uniquely named with the name of the gene following by a successive number according with the position in the gene and species identification (first all the cattle SNP, then the buffalo SNP). Five SNP were overlapping among *B. bubalis* and *B. taurus* (SCARB1.1 with SCARB1.2, SCARB1.5 with SCARB1.6, SCARB1.7 with SCARB1.8, BCMO1.10 with BCMO1.11). The number of SNP detected in the coding regions varied from 16 to 26 in the 3 species, with 5 overlapping SNP between *B. taurus* and *B. indicus*. This is due to the high similarity among the 2 species that are often crossed to improve the production. Therefore, all SNP designed on cattle were considered for both breed crosses and *B. indicus* breeds. The genotypes with the Indian test panel cattle (Supplemental Table 4a, https://doi.org/10.3168/jds.2019-16361) reported that BCMO1.4 was not successfully genotyped. The other SNP showed a call rate ranging from 75 to 100%, with 29 SNP (12 for the *BCO2* gene, 15 for the *SCARB1* gene, and 2 for the *BCMO1* gene) with a call rate ≥0.90% in all the considered cattle breeds and crosses. For the buffalo breeds (Supplemental Table 4b, https://doi.org/10.3168/jds.2019-16361), 17 out of the 24 SNP designed for buffalo were genotyped with a call rate ≥90% in all the breeds. These SNP were 5 for *BCO2*, 7 for *SCARB1*, and 5 for the *BCMO1* genes.

**Table 1:** Sequenom panel coordinates for the SNP that targeted cattle (*Bos taurus* and *Bos indicus*) are made based on the UMD3.1 (https://www.ncbi.nlm.nih.gov/assembly/GCF_000003055.6) reference genome^1^

Genomic coordinate	Sequenom SNP	Species	Effect	dbSNP
15:g.22841716T>G	BCO2.1	*B. indicus*	Synonymous	—
15:g.22877552G>A	BCO2.2	*B. taurus*	Stop	rs109226280
15:g.22878937G>A	BCO2.3	*B. taurus*	Nonsynonymous	rs445248588
15:g.22886751A>T	BCO2.4	*B. taurus*	Stop	rs475149853
15:g.22886781G>A	BCO2.5	*B. indicus*	Nonsynonymous	—
15:g.22887879G>A	BCO2.6	*B. indicus*	Nonsynonymous	—
15:g.22902367G>A	BCO2.7	*B. taurus*	Synonymous	rs468029187
15:g.22903083C>T	BCO2.8	*B. taurus*	Nonsynonymous	rs463702615
15:g.22903195A>G	BCO2.9	*B. indicus*	Synonymous	—
15:g.22904160C>T	BCO2.10	*B. indicus*	Nonsynonymous	—
15:g.22904161A>T	BCO2.11	*B. indicus*	Nonsynonymous	—
15:g.22905346T>C	BCO2.12	*B. indicus*	Nonsynonymous	—
17:g.53180716C>G	SCARB1.1^1^	*B. taurus*	Upstream variant	rs211588107
17:g.53180716C>G	SCARB1.2^1^	*B. indicus*	Upstream variant	—
17:g.53181068C>G	SCARB1.3	*B. indicus*	Nonsynonymous	—
17:g.53229545A>C	SCARB1.4	*B. indicus*	Nonsynonymous	—
17:g.53237728A>G	SCARB1.5^2^	*B. taurus*	Synonymous	rs210238050
17:g.53237728A>G	SCARB1.6^2^	*B. indicus*	Synonymous	—
17:g.53237845T>C	SCARB1.7^3^	*B. taurus*	Synonymous	rs210829935
17:g.53237845T>C	SCARB1.8^3^	*B. indicus*	Synonymous	—
17:g.53242822C>A	SCARB1.9	*B. taurus*	Synonymous	rs377844543
17:g.53242843C>T	SCARB1.10	*B. indicus*	Synonymous	—
17:g.53242852C>T	SCARB1.11	*B. indicus*	Synonymous	—
17:g.53242906C>T	SCARB1.12	*B. taurus*	Synonymous	rs478582082
17:g.53245654G>A	SCARB1.13^4^	*B. taurus*	Synonymous	rs211138720
17:g.53245654G>A	SCARB1.14^4^	*B. indicus*	Synonymous	—
17:g.53258090A>G	SCARB1.15	*B. indicus*	Synonymous	—
17:g.53263896C>T	SCARB1.16	*B. indicus*	Synonymous	—
18:g.7930930C>T	BCMO1.1	*B. taurus*	Upstream gene variant	rs110137311
18:g.7938090G>A	BCMO1.2	*B. taurus*	Nonsynonymous/stop	rs210669227
18:g.7938092G>A	BCMO1.3	*B. indicus*	Synonymous	—
18:g.7942847T>C	BCMO1.4	*B. indicus*	Synonymous	—
18:g.7942924G>C	BCMO1.5	*B. indicus*	Nonsynonymous	—
18:g.7944544C>A	BCMO1.6	*B. taurus*	Synonymous	np
18:g.7944547G>A	BCMO1.7	*B. indicus*	Synonymous	—
18:g.7944577C>T	BCMO1.8	*B. indicus*	Synonymous	—
18:g.7947135G>C	BCMO1.9	*B. indicus*	Nonsynonymous	—
18:g.7947229C>T	BCMO1.10^5^	*B. taurus*	Synonymous	rs381162140
18:g.7947229C>T	BCMO1.11^5^	*B. indicus*	Synonymous	—
18:g.7947242G>A	BCMO1.12	*B. taurus*	Nonsynonymous	rs209658446
18:g.7949278A>G	BCMO1.13	*B. indicus*	Synonymous	—
18:g.7949326T>C	BCMO1.14	*B. indicus*	Synonymous	—
18:g.7949381A>G	BCMO1.15	*B. taurus*	Nonsynonymous	rs444976967
18:g.7962378C>G	BCMO1.16	*B. indicus*	Nonsynonymous	—
jcf7180021617284:g.785353G>A	BCO2.13	*Bubalus bubalis*	Synonymous	—
jcf7180021617284:g.785395C>T	BCO2.14	*B. bubalis*	Synonymous	—
jcf7180021617284:g.787642T>C	BCO2.15	*B. bubalis*	Nonsynonymous	—
jcf7180021617284:g.788450C>T	BCO2.16	*B. bubalis*	Synonymous	—
jcf7180021617284:g.845577G>A	BCO2.17	*B. bubalis*	Synonymous	—
jcf7180021617284:g.847449G>A	BCO2.18	*B. bubalis*	Nonsynonymous	—
jcf7180021616390:g.1200693C>T	SCARB1.17	*B. bubalis*	Synonymous	—
jcf7180021616390:g.1226834G>T	SCARB1.18	*B. bubalis*	Nonsynonymous	—
jcf7180021616390:g.1226890G>C	SCARB1.19	*B. bubalis*	Synonymous	—
jcf7180021616390:g.1226924A>G	SCARB1.20	*B. bubalis*	Nonsynonymous	—
jcf7180021616390:g.1226944G>A	SCARB1.21	*B. bubalis*	Synonymous	—
jcf7180021616390:g.1231985C>T	SCARB1.22	*B. bubalis*	Nonsynonymous	—
jcf7180021616390:g.1235067C>T	SCARB1.23	*B. bubalis*	Synonymous	—
jcf7180021616390:g.1235151G>A	SCARB1.24	*B. bubalis*	Synonymous	—
jcf7180021616390:g.1283516A>C	SCARB1.25	*B. bubalis*	Synonymous	—
jcf7180021615735:g.3038221G>T	BCMO1.17	*B. bubalis*	Missense	—
jcf7180021615735:g.3038243C>T	BCMO1.18	*B. bubalis*	Synonymous	—
jcf7180021615735:g.3040767G>T	BCMO1.19	*B. bubalis*	Synonymous	—
jcf7180021615735:g.3051607C>T	BCMO1.20	*B. bubalis*	Synonymous	—
jcf7180021615735:g.3062829G>C	BCMO1.21	*B. bubalis*	Nonsynonymous	—
jcf7180021615735:g.3062850G>A	BCMO1.22	*B. bubalis*	Synonymous	—
jcf7180021615735:g.3062908G>A	BCMO1.23	*B. bubalis*	Nonsynonymous	—
jcf7180021615735:g.3068901A>G	BCMO1.24	*B. bubalis*	Synonymous	—

SNP independently detected but overlapping in both *B. taurus* and *B. indicus* are reported with the same apical number in the Sequenom column.

### Genotyping of the BC Samples and Association Analyses

***LD***. The genotyping of the animals was successful in at least one breed for all the SNP except for BCMO1.1 (cattle) and BCMO1.17 SNP (buffalo). The number of SNP successfully genotyped for cattle breeds ranged from 42 (Gir) to 31 (Tharparkar) with the combined cattle breeds having 28 high quality SNP in common. In buffalo, the number of high-quality SNP ranged from 22 (Jafarabadi, Mehsana, Pandharpuri) to 20 (Murrah, Surti), whereas the combined buffalo breeds had 20 SNP in common that were successfully genotyped. The pairwise analyses performed on cattle and buffalo SNP revealed a low number of SNP in high LD (Supplemental Tables S5a and S5b, https://doi.org/10.3168/jds.2019-16361). For cattle, as expected, mostly of the duplicated SNP in cattle and buffalo are in strong LD for most of the breeds. The *BCO2* gene had BCO2.2 and BCO2.6 in strong linkage in the Tharparkar breed, whereas the *SCARB1* gene has several SNP in strong LD, where SCARB1.13 and SCARB1.14 were in strong LD in all the considered breeds. The *BCMO1* gene had BCMO1.8 and BCMO1.9 that are in high LD within Gir, Sahiwal, and Jersey breeds. As for the buffalo breeds, only the *SCARB1* gene reported 4 SNP (SCARB1.5, SCARB1.6, SCARB1.7, and SCARB1.8) in strong LD in all the breeds.

***BC and Association Analyses***. An initial breed and species comparison demonstrated differences in BC level in milk among cattle and buffalo with buffalo showing a lower BC content than cattle (Table 2). This may be because buffalo can convert some portion of BC directly in vitamin A (Ullah et al., 2017).

**Table 2:** Number and mean β-carotene (BC) concentration in milk

Species	Breed	No. of samples	BC mean (μg/100 mL)	SE
Cattle	Combined	2,291	4.41	0.11
	Holstein cross	492	6.16	0.38
	Jersey cross	512	3.90	0.16
	Sahiwal	392	4.34	0.24
	Tharparkar	481	4.04	0.18
	Gir	414	3.50	0.17
Buffalo	Combined	2,242	4.33	0.11
	Jaffrabadi	458	5.50	0.26
	Murrah	470	4.71	0.25
	Pandharpuri	412	3.35	0.19
	Mehsana	489	4.31	0.28
	Surti	413	3.61	0.23

The specific linear models used for association analyses for each breed and species are shown in Tables 3 and 4. For each model, adding different SNP to the linear models increased the R^2^ value by a limited amount in most cases, suggesting that variation in BC content was mostly affected by environmental effects. The highest R^2^ was seen for models for Sahiwal cattle and the lowest R^2^ for models for Tharparkar cattle. For buffalo the lowest R^2^ was produced in the combined analyses, whereas the highest was produced by the Murrah breed.

**Table 3:** Fixed effects included in the linear models used to analyze each cattle breed

Breed	Model^1^	R^2^
Combined	Breed + place (breed) + farmer (place x breed)	0.566
	All common SNP^2^	0.577
	Nonsynonymous/stop^3^	0.57
	*P* < 0.3 SNP^4^ (11 SNP)	0.573
Holstein cross	Farmer + place	0.545
	All SNP^5^	0.574
	Nonsynonymous/stop	0.551
	*P* < 0.3 SNP (10 SNP)	0.558
Jersey cross	Farmer + place + yield	0.807
	All SNP	0.861
	Nonsynonymous/stop	0.815
	*P* < 0.3 SNP (12 SNP)	0.848
Gir	Farmer + place + lactation + yield	0.58
	All SNP	0.642
	Nonsynonymous/stop	0.608
	*P* < 0.3 SNP (9 SNP)	0.621
Sahiwal	Farmer + place + lactation + yield	0.871
	All SNP	0.913
	Nonsynonymous/stop	0.893
	*P* < 0.3 SNP (13 SNP)	0.898
Tharparkar	Farmer + place + lactation + yield	0.112
	All SNP	0.155
	Nonsynonymous/stop	0.128
	*P* < 0.3 SNP (7 SNP)	0.133

1 The initial model does not include SNP. The following models include the initial model plus different sets of SNP.

2 Model including as all SNP with call rate ≥90% for all 5 breeds in addition to fixed effects.

3 Model including only SNP that code for nonsynonymous and stop codons in addition to fixed effects.

4 Model including only SNP with *P*-value ≤0.3 in addition to fixed effects.

5 Model including all SNP with call rate ≥90% in addition to fixed effects.

**Table 4:** Fixed effects included in the linear models used to analyze each buffalo breed

Breed	Model^1^	R^2^
Combined	Breed + batch(breed) + place(batch x breed)	0.336
	All common SNP^2^	0.346
	Nonsynonymous/stop^3^	0.341
	*P* < 0.3 SNP^4^ (8 SNP)	0.344
Jafarabadi	Batch + farmer + place + lactation + yield	0.475
	All SNP^5^	0.516
	Nonsynonymous/stop	0.498
	*P* < 0.3 SNP (6 SNP)	0.504
Mehsana	Batch + farmer + place + lactation	0.464
	All SNP	0.469
	Nonsynonymous/stop	0.492
	*P* < 0.3 SNP (6 SNP)	0.503
Murrah	Batch + farmer + place + lactation + yield	0.873
	All SNP	0.902
	Nonsynonymous/stop	0.872
	*P* < 0.3 SNP (4 SNP)	0.886
Pandharpuri	Batch + farmer + place	0.84
	All SNP	0.88
	Nonsynonymous/stop	0.885
	*P* < 0.3 SNP (9 SNP)	0.916
Surti	Batch + farmer + place + lactation + yield	0.408
	All SNP	0.483
	Nonsynonymous/stop	0.431
	*P* < 0.3 SNP (8 SNP)	0.459

1 The initial model does not include SNP. The following models include the initial model plus different sets of SNP.

2 Model including as all SNP with call rate ≥90% for all 5 breeds in addition to fixed effects.

3 Model including only SNP that code for nonsynonymous and stop codons in addition to fixed effects.

4 Model including only SNP with *P*-value ≤0.3 in addition to fixed effects.

5 Model including all SNP with call rate ≥90% in addition to fixed effects.

The allele substitution effects were estimated with the complete linear model for each breed including the SNP with *P* < 0.3 as removing SNP with *P* > 0.3 did not change the overall type I error rate and was a good compromise for acceptance and rejection. In Tables 5 and 6, allele substitution effects and the recommended alleles to select for each SNP are presented, as well as the gene-wise *F*-test performed to determine which genes had a significant effect on BC concentration as a whole. All breeds of cattle and buffalo showed SNP with *P*-value <0.3 except for the Mehsana buffalo breed. Even though all SNP with *P*-value <0.3 were included in the final analyses, attention should be centered on SNP that are significant at 0.05. This includes 23 different SNP for cattle (13 for the combined breeds, 9 for Holstein, 10 for Jersey, 11 for Gir, 13 for Sahiwal, and 4 for Tharparkar; Table 5) and 17 different SNP for buffalo (10 for the combined breeds, 7 for Jafarabadi, 6 for Murrah, 3 for Pandharpuri, and 9 for Surti; Table 6). These SNP possibly represent promising candidates for future selective breeding of improved BC production, but larger sample sizes are needed to confirm this.

**Table 5:** Cattle SNP with *P*-values ≤0.30 and *F*-tests for significant SNP in each gene^1^

Breed	Population mean^2^	Model	SNP				Gene effect (μg/100 mL)	Total effect^3^ (μg/100 mL; SE)
Combined	4.41 (0.11)	Breed + place (breed) + farmer (place × breed)						17.38 (4.31) *P* < 0.0001
		BCMO1	BCMO1.3	BCMO1.11	BCMO1.15			
		Allele substitution effect (SE; μ/100 mL)	–0.536 (0.38)	1.286 (0.47)	–1.937 (1.08)		7.57 (2.48)	
		*P*-value	0.16	0.006	0.007		0.002^a^	
		Recommended allele	A	T	A			
		Frequency of recommended allele	0.07	0.04	0.99			
		BCO2	BCO2.2	BCO2.4	BCO2.5	BCO2.12		
		Allele substitution effect (SE; μ/100 mL)	1.212 (0.87)	–0.965 (0.92)	0.368 (0.32)	–0.399 (0.31)	5.88 (2.84)	
		*P*-value	0.16	0.3	0.25	0.2	0.04	
		Recommended allele	G	A	G	C		
		Frequency of recommended allele	0.63	0.99	0.88	0.24		
		SCARB1	SCARB1.2	SCARB1.3	SCARB1.8			
		Allele substitution effect (SE; μ/100 mL)	0.516 (0.32)	0.715 (0.39)	–0.745 (0.37)		3.98 (1.73)	
		*P*-value	0.11	0.07	0.05		0.02	
		Recommended allele	C	C	T			
		Frequency of recommended allele	0.7	0.54	0.45			
Holstein cross	6.16 (0.38)	Farmer + place						30.62 (10.04) *P* < 0.002
		BCMO1	BCMO1.5	BCMO1.12	BCMO1.16			
		Allele substitution effect (SE; μ/100 mL)	–3.40 (1.80)	0.80 (0.57)	–2.76 (2.02)		14.02 (5.64)	
		*P*-value	0.06	0.16	0.17		0.01	
		Recommended allele	G	G	G			
		Frequency of recommended allele	0.98	0.85	0.01			
		BCO2	BCO2.1	BCO2.2	BCO2.6	BCO2.11		
		Allele substitution effect (SE; μ/100 mL)	1.80 (1.45)	2.85 (1.59)	–1.82 (1.68)	1.94 (1.81)	16.58 (8.18)	
		*P*-value	0.21	0.07	0.28	0.29	0.04	
		Recommended allele	T	G	A	G		
		Frequency of recommended allele	0.93	0.99	0.12	0.97		
Jersey cross	3.90 (0.16)	Farmer + place + yield						31.06 (14.02) *P* < 0.0001
		BCO2	BCO2.1	BCO2.3	BCO2.4	BCO2.6		
		Allele substitution effect (SE; μ/100 mL)	–1.55 (1.07)	–2.66 (1.71)	–1.09 (0.89)	1.36 (0.98)	13.32 (9.30)	
		*P*-value	0.15	0.13	0.21	0.12	0.01	
		Recommended allele	G	A	A	G		
		Frequency of recommended allele	0.28	0.02	0.63	0.73		
		SCARB1	SCARB1.3	SCARB1.5	SCARB1.10	SCARB1.16		
		Allele substitution effect (SE; μ/100 mL)	2.31 (1.29)	1.18 (0.58)	3.18 (0.92)	–2.19 (1.56)	17.74 (4.72)	
		*P*-value	0.07	0.03	0.0009	0.17	0.0003	
		Recommended allele	C	G	T	C		
		Frequency of recommended allele	0.68	0.58	0.14	0.99		
Gir	3.50 (0.17)	Farmer + place + lactation + yield						33.86 (8.76) *P* < 0.0001
		BCMO1	BCMO1.2	BCMO1.3	BCMO1.8			
		Allele substitution effect (SE; μ/100 mL)	–4.72 (3.18)	–1.03 (0.64)	–1.24 (0.54)		14.1 (6.66)	
		*P*-value	0.14	0.11	0.02		0.04	
		Recommended allele	A	A	C			
		Frequency of recommended allele	0.002	0.1	0.8			
		BCO2	BCO2.5	BCO2.9	BCO2.10	BCO2.12		
		Allele substitution effect (SE; μ/100 mL)	2.39 (1.01)	–1.60 (0.74)	–3.13 (1.20)	–1.48 (0.90)	17.12 (5.40)	
		*P*-value	0.02	0.03	0.01	0.1	0.002	
		Recommended allele	G	A	C	C		
		Recommended allele frequency	0.88	0.89	0.09	0.04		
		SCARB1	SCARB1.5					
		Allele substitution effect (SE; μ/100 mL)	1.28 (0.50)				2.56 (1.00)	
		*P*-value	0.01				0.01	
		Recommended allele	G					
		Frequency of recommended allele	0.97					
Sahiwal	4.34 (0.24)	Farmer + place + lactation + yield						39.06 (10.99) *P* < 0.0006
		BCMO1	BCMO1.8	BCMO1.12	BCMO1.14			
		Allele substitution effect (SE; μ/100 mL)	0.91 (0.78)	–2.48 (1.60)	0.93 (0.88)		8.64 (3.84)	
		*P*-value	0.25	0.12	0.29		0.03	
		Recommended allele	T	A	T			
		Frequency of recommended allele	0.32	0.02	0.30			
		BCO2	BCO2.9	BCO2.11	BCO2.12			
		Allele substitution effect (SE; μ/100 mL)	–1.43 (1.09)	–1.86 (1.03)	–1.45 (0.80)		9.48 (3.98)	
		*P*-value	0.19	0.07	0.07		0.02	
		Recommended allele	A	A	C			
		Frequency of recommended allele	0.92	0.06	0.24			
		SCARB1	SCARB1.5	SCARB1.7	SCARB1.10	SCARB1.16		
		Allele substitution effect (SE; μ/100 mL)	–1.67 (0.10)	–2.00 (1.15)	–0.58 (0.48)	–6.21 (3.13)		
		*P*-value	0.1	0.08	0.23	0.05	0.02	
		Recommended allele	A	C	C	C		
		Frequency of recommended allele	0.04	0.52	0.78	0.99		
Tharparkar	4.04 (0.18)	Farmer + place + lactation + yield						1.78 (1.025) *P* < 0.08
		BCMO1	BCMO1.9					
		Allele substitution effect (SE; μ/100 mL)	0.48 (0.39)				0.96 (0.78)	
		*P*-value	0.23				0.23	
		Recommended allele	C					
		Recommended allele frequency	0.19					
		BCO2	BCO2.1					
		Allele substitution effect (SE; μ/100 mL)	–0.41 (0.31)				0.82 (0.62)	
		*P*-value	0.18				0.18	
		Recommended allele	G					
		Frequency of recommended allele	0.6					

a *P*-value for gene-wide effect.

1 *BCM01, SGARB1*, and *BCO2* are the gene symbols.

2 Mean β-carotene content for the breed (SE; μg/100 mL).

3 Represents EBV of an animal homozygous for all favorable alleles.

**Table 6:** Buffalo SNP with *P* < 0.30 and *F*-tests for significant SNP in each gene^1,2^

Breed	Population mean^3^	Model	SNP				Gene effect (μ/100 mL)	Total effect^4^ μg/100 mL; SE)
Combined	4.33 (0.11)	Breed + batch (breed) + place (batch × breed)						4.72 (1.046) *P* < 0.0001
		BCMO1	BCMO1.21	BCMO1.24				
		Allele substitution effect (SE; μ/100 mL)	0.17 (0.15)	0.37 (0.23)			1.08 (0.52)	
		*P*-value	0.25	0.1			0.04^a^	
		Recommended allele	C	A				
		Frequency of recommended allele	0.58	0.56				
		BCO2	BCO2.16	BCO2.18				
		Allele substitution effect (SE; μ/100 mL)	0.45 (0.28)	0.38 (0.17)			1.62 (0.66)	
		*P*-value	0.1	0.03			0.01	
		Recommended allele	T	A				
		Frequency of recommended allele	0.07	0.84				
		SCARB1	SCARB1.17	SCARB1.19	SCARB1.20			
		Allele substitution effect (SE; μ/100 mL)	0.23 (0.16)	0.35 (0.19)	–0.35 (0.27)		1.96 (0.66)	
		*P*-value	0.16	0.06	0.2		0.003	
		Recommended allele	T	C	G			
		Frequency of recommended allele	0.55	0.46	0.72			
Jafarabadi	5.5 (0.26)	Batch + farmer + place + lactation + yield						
		BCMO1	BCMO1.21	BCMO1.24				7.84 (2.31) *P* < 0.0008
		Allele substitution effect (SE; μ/100 mL)	1.40 (0.53)	1.38 (0.78)	5.56 (1.58)			
		*P*-value	0.008	0.08			0.0005	
		Recommended allele	C	A				
		Frequency of recommended allele	0.62	0.88				
		BCO2	BCO2.14					
		Allele substitution effect (SE; μ/100 mL)	0.55 (0.49)				1.10 (0.98)	
		*P*-value	0.26				0.26	
		Recommended allele	T					
		Frequency of recommended allele	0.43					
		SCARB1	SCARB1.19					
		Allele substitution effect (SE; μ/100 mL)	0.59 (0.51)				1.18 (1.02)	
		*P*-value	0.24				0.24	
		Recommended allele	C					
		Frequency of recommended allele	0.54					
Murrah	4.71 (0.25)	Batch + farmer + place + lactation + yield						
		BCMO1	BCMO1.22	BCMO1.24				
		Allele substitution effect (SE; μ/100 mL)	0.44 (0.27)	0.41 (0.36)			1.7 (0.94)	10.64 (4.68) *P* < 0.02
		*P*-value	0.1	0.25			0.07	
		Recommended allele	C	A				
		Frequency of recommended allele	0.47	0.84				
		SCARB1	SCARB1.22	SCARB1.24				
		Allele substitution effect (SE; μ/100 mL)	–2.06 (1.11)	2.41 (1.12)			8.94 (4.44)	
		*P*-value	0.07	0.03			0.05	
		Recommended allele	C	A				
		Frequency of recommended allele	0.31	0.70				
Pandharpuri	3.35 (0.19)	Batch + farmer + place + lactation						
		BCMO1	BCMO1.19	BCMO1.24				
		Allele substitution effect (SE; μ/100 mL)	–1.83 (1.31)	–3.10 (2.16)				9.86 (5.48) *P* < 0.08
		*P*-value	0.17	0.16				
		Recommended allele	T	G				
		Frequency of recommended allele	0.15	0.04				
Surti	3.61 (0.23)	Farmer + place + lactation + yield						
		BCMO1	BCMO1.20	BCMO1.22	BCMO1.23	BCMO1.24		33.71 (12.30) *P* < 0.007
		Allele substitution effect (SE; μ/100 mL)	–1.41 (1.03)	0.85 (0.69)	9.10 (5.43)	–1.38 (0.84)	22.67 (11.66)	
		*P*-value	0.17	0.22	0.1	0.1	0.05	
		Recommended allele	C	A	A	G		
		Frequency of recommended allele	0.92	0.27	0.001	0.15		
		BCO2	BCO2.16					
		Allele substitution effect (SE; μ/100 mL)	2.88 (1.17)				6.76 (2.38)	
		*P*-value	0.009				0.005	
		Recommended allele	T					
		Frequency of recommended allele	0.07					
		SCARB1	SCARB1.20					
		Allele substitution effect (SE; μ/100 mL)	–2.07 (0.88)				4.14 (1.76)	
		*P*-value	0.02				0.02	
		Recommended allele	G					
		Frequency of recommended allele	0.7					

a *P*-value for gene-wide effect.

1 No SNP with *P*-value ≤0.3 were found for Mehsana; therefore, no SNP were included in these analyses.

2 *BCM01, SCARP1*, and *BCO2* are the gene symbols.

3 Mean β-carotene content for the breed (SE; μg/100 mL).

4 Represents EBV of an animal homozygous for all favorable alleles.

The analyses performed identified multiple markers in the *BCO2, BCMO1*, and *SCARB1* genes for the population of cattle and buffalo investigated. These results enrich what has been detected for *BCO2*, where one marker was identified for BC concentrations in cow milk (Berry et al., 2009).

In cattle, the significant SNP with the largest allele substitution effect was SCARB1.16 for the Sahiwal breed with *P*-value <0.05 and an allele substitution effect of –6.21 ± 3.13 μg of BC/100 mL of milk; for this SNP, animals should be selected for the C allele. In buffalo, SCARB1.20 of Surti animals was the significant SNP with the largest allele substitution effect of –2.07 ± 0.88 μg of BC/100 mL of milk, in this case selection should be made for the G allele. It is important to note that there are 3 SNP with *P* < 0.05 for the combined cattle (BCMO1.11, BCMO1.15, SCARB1.8) analyses and only one for buffalo (BCO2.18). These markers might represent an important tool for selection for cattle and buffaloes when breed proportions are unknown because they are significant when the breeds of each species included in this research are analyzed simultaneously. These SNP may also be particularly useful when the breed of the animal is unknown or in case of crossbreeds. As for the single breeds, SNP with a significant *P*-value included Jersey crosses (SCARB1.10), Gir (BCMO1.8, BCO2.2, BCO2.5, BCO2.9, and BCO2.10), and Sahiwal (Scarb1.16) for cattle; and Jafarabadi (BCMO.21), Murah (SCARB1.24), and Surti (BCO2.16 and SCARB1.20) for buffalo. Finally, there are significant cases such as the *BCMO1* gene for Gir cattle and Jafarabadi where several markers were in LD within the gene and therefore it might be beneficial to select for a specific haplotype in that gene.

In the Bayesian approach, inferences are based on posterior probabilities. The Bayes A prior assumes a *t* distribution centered at zero for the effects of all loci, and so each locus is a priori equally likely to be positive or negative. On the other hand, Bayes Cπ prior assumes the SNP effect is null with probability π or has a normal distribution with probability 1 – π for all loci, where the probability π is treated as an unknown with a uniform prior. Thus, in a Bayes Cπ analysis, the posterior probability (**PP**) that a locus has a non-null effect can be taken as evidence of an association of the SNP with the trait.

Thus, PP that deviate from this prior probability of 0.5 can be taken as evidence of an association of the SNP with the trait. Table 7 gives SNP that had PP greater than 0.8, 0.7, or 0.5 (showing all SNP) for being positive or being negative, together with the posterior mean for the difference between the most favorable and the least favorable genotypes involving these SNP.

**Table 7:** Significant SNP for cattle and buffalo and total effect (STD) using a Bayesian approach (Bayes A)

Breed^1^	Gene	Posterior probability (PP) > 0.8		PP > 0.7		PP > 0.5^2^	
		SNP	Total effect^3^ (μg/100 mL; SD)	SNP	Total effect (μg/100 mL; SD)	SNP	Total effect (μg/100 mL; SD)
Cattle combined	*BCMO1*	2, 3, 5, 11, 15	13.09 (3.74)	2, 3, 5, 11, 15	15.40 (4.26)	2, 3, 5, 6, 7, 10, 11, 15	19.13 (5.73)
	*BCO2*	2, 5,		2, 4, 5, 12		1, 2, 4, 5, 6, 7, 8, 11, 12	
	*SCARB1*	2, 3, 8, 11		2, 3, 8, 11, 13		2, 3, 4, 8, 9, 10, 11, 13, 14, 16	
Holstein cross	*BCMO1*	5, 12	7.70 (7.08)	3, 5, 8, 10, 11, 12, 16	16.64 (6.07)	2, 3, 5, 6, 7, 8, 10, 11, 12, 15, 16	27.78 (11.71)
	*BCO2*	2		1, 2		1, 2, 4, 5, 6, 7, 8, 11	
	*SCARB1*	3		3		1, 2, 3, 5, 7, 8, 10, 13, 14, 16	
Jersey cross	*BCMO1*	None	6.85 (3.28)	15	9.80 (4.22)	2, 3, 5, 7, 8, 9, 10, 11, 12, 14, 15, 16	19.29 (9.55)
	*BCO2*	4		1, 3, 4		1, 2, 3, 4, 5, 6, 7, 8, 9, 10, 11, 12	
	*SCARB1*	3, 5, 10		3, 5, 10		1, 2, 3, 5, 6, 7, 8, 9, 10, 11, 12, 13, 14, 15, 16	
Gir	*BCMO1*	3, 16	7.10 (3.53)	3, 8, 16	11.64 (4.76)	2, 3, 5, 7, 8, 9, 10, 11, 12, 13, 14, 16	19.27 (8.12)
	*BCO2*	5, 9, 10		5, 8, 9, 10, 12		1, 2, 3, 5, 6, 8, 9, 10, 11, 12	
	*SCARB1*	5		4, 5, 11, 15		1, 2, 3, 4, 5, 6, 7, 8, 10, 11, 12, 13, 14, 15, 16	
Sahiwal	*BCMO1*	None	3.32 (1.97)	7, 9, 12	9.19 (4.48)	2, 3, 5, 7, 8, 9, 10, 11, 12, 14, 16	17.31 (8.86)
	*BCO2*	11, 12		6, 9, 11, 12		1, 2, 4, 5, 6, 8, 9, 10, 11, 12	
	*SCARB1*	10		5, 10, 13		1, 2, 3, 5, 6, 7, 8, 9, 10, 11, 13, 14, 16	
Tharparkar	*BCMO1*	7	4.61 (2.15)	7, 11	5.96 (2.78)	3, 5, 6, 7, 8, 9, 10, 11, 15	12.71 (7.20)
	*BCO2*	5		5		1, 4, 5, 6, 8, 11, 12	
	*SCARB1*	2, 11		2, 11		2, 3, 4, 6, 8, 10, 11, 13, 14, 16	
Buffalo combined	*BCMO1*	21, 24	5.99 (1.67)	18, 21, 24	7.79 (2.37)	18, 20, 21, 22, 23, 24	9.49 (3.78)
	*BCO2*	16, 17, 18		16, 17, 18		14, 15, 16, 17, 18	
	*SCARB1*	17, 19, 20, 21		17, 19, 20, 21, 22, 23, 24		17, 18, 19, 20, 21, 22, 23, 24, 25	
Jaffarbadi	*BCMO1*	21, 22, 24	5.50 (1.73)	21, 22, 24	6.86 (2.03)	18, 19, 20, 21, 22, 24	10.60 (5.96)
	*BCO2*	None		14		13, 14, 16, 17, 18	
	*SCARB1*	19		16, 19		17, 18, 19, 20, 21, 22, 23, 24, 25	
Murrah	*BCMO1*	21, 24	5.98 (1.48)	18, 21, 24	8.45 (2.38)	18, 20, 21, 22, 24	11.44 (4.81)
	*BCO2*	16, 18		16, 18		14, 16, 17, 18	
	*SCARB1*	None		19, 24		17, 18, 19, 20, 21, 22, 23, 24, 25	
Pandharpuri	*BCMO1*	24	5.49 (2.93)	21, 24	11.62 (4.38)	18, 19, 20, 21, 22, 23, 24	15.45 (8.28)
	*BCO2*	18		14, 18		13, 14, 15, 16, 17, 18	
	*SCARB1*	25		9, 19, 21, 22, 23		17, 18, 19, 20, 21, 22, 23, 24, 25	
Surti	*BCMO1*	18, 24	7.60 (2.92)	18, 20, 22, 24	12.25 (4.28)	18, 20, 21, 22, 23, 24	16.16 (5.93)
	*BCO2*	16		16		14, 16, 17, 18	
	*SCARB1*	19		17, 19, 20, 25		17, 18, 19, 20, 21, 22, 23, 24, 25	

1 No SNP with *P*-value ≤0.3 were found for Mehsana; therefore, no SNP were included in these analyses.

2 Includes all SNP with *P*-value ≤0.3.

3 Represents EBV of an animal homozygous for all favorable alleles.

In several cases, the 2 analyses suggested the same markers were significant. However, it is often misleading to just compare results from the different approaches as they should be expected to differ for several reasons, but we include this discussion for completeness of these results. Estimates (posterior means) of the marker effects, obtained from the Bayesian approach, ranged from 0.01 to 2.02 (data not shown). Means for total gain, generally, were similar or smaller than those found with the frequentist approach. Interestingly, even though the frequentist approach is not directly comparable with the Bayesian methods, the number of SNP with important effects was similar; in many cases the different sets of SNP in the 2 approaches with important effects overlapped. When comparing the results of the frequentist approach and the most stringent threshold of both Bayesian approaches, 6 SNP overlapped for the combined cattle breeds. For the buffalo combined breeds, 7 SNP overlapped across the 3 analyses performed. The biggest difference was found in the Jersey cattle breed for which the Bayesian analyses found 5 fewer important SNP than were found with the frequentist approach and total gain with the frequentist approach was nearly 45 μg/100 mL higher when compared with the results of the Bayesian approach.

In the combined buffalo breeds the frequentist approach found 7 significant SNP, and the Bayesian approach with a Bayes A prior and a PP > 0.7 found 13. Results from the Bayes Cπ analyses showed a different trend. The SNP that had non-null effects with a PP greater than 0.0 (showing all SNP), 0.1, and 0.2 are given in Table 8 for all breeds except for the Surti and Pandharpuri breeds, which showed markers with important effects with PP > 0.4 and PP > 0.5 (Table 8), along with the posterior means for total gain in BC content in milk of animals that are homozygous for the favorable alleles at these SNP. Some buffalo breeds tended to show higher total gains than any of the cattle breeds when the Bayes Cπ prior was used to analyze the data, exhibiting an opposite trend to both the Bayes A and frequentist analyses that showed cattle breeds having higher total gains when compared with buffalo breeds. For the analyses with the Bayes Cπ prior, the breeds with the overall highest total gain were the buffalo breeds Pandharpuri and Surti with 10.42 ± 7.33 and 9.43 ± 5.67 μg/100 mL, respectively, for PP > 0.0. For cattle, Holstein-crossed animals showed the highest total gain with 6.34 ± 7.20 μg/100 mL. Another result is that only buffalo breeds (Pandharpuri, Surti, and Jafarabadi) showed markers that had PP higher than 0.2. and that reached 0.8 in the case of Jafarabadi. These 2 breeds, however, had only 213 (Pandharpuri) and 388 (Surti) observations, and as a result, the posterior mean of π was 0.51 for Pandharpuri and 0.55 for Surti, which are very close to the prior mean of 0.5. Thus, posterior means in the Bayes Cπ analyses for total gain for these 2 breeds were close to but lower than those from the Bayes A analyses, which implicitly has a π of 0.0. When the breeds were combined within cattle (2,290 observations) and buffalo (2,238 observations), the posterior mean of π was 0.88 for cattle and was 0.87 for buffalo, which implies that a large proportion of the SNP had no association with the trait. Thus, posterior means in the Bayes Cπ analysis for total gain in cattle and buffalo were much lower than those from the Bayes A analyses, but they were higher for cattle than for buffalo as in the Bayes A analyses.

**Table 8:** Significant SNP for cattle and buffalo and total effect (SD) using a Bayesian approach (Bayes Cπ)

Breed^1^	Gene	Posterior probability (PP) > 0.0^2^		PP > 0.1^2^		PP > 0.2^2^	
		SNP	Total effect^3^ (μg/100 mL; SD)	SNP	Total effect^3^ (μg/100 mL; SD)	SNP	Total effect^3^ (μg/100 mL; SD)
Cattle combined	*BCMO1*	2, 3, 5, 6, 7, 10, 11, 15	3.13 (3.52)	2, 3, 5, 6, 10, 11, 15	2.89 (3.21)	10, 11, 15	1.48 (1.75)
	*BCO2*	1, 2, 4, 5, 6, 7, 8, 11, 12		2, 4		None	
	*SCARB1*	2, 3, 4, 8, 9, 10, 11, 13, 14, 16		4, 8, 9, 13, 14		None	
Holstein cross	*BCMO1*	2, 3, 5, 6, 7, 8, 10, 11, 12, 15, 16	6.34 (7.20)	2, 3, 5, 6, 7, 8, 10, 11 12, 15 16	6.32 (7.18)	3, 5, 7, 8, 10, 11, 16	3.90 (4.63)
	*BCO2*	1, 2, 4, 5, 6, 7, 8, 11		1, 2, 3, 4, 5, 6, 7, 8, 10, 11		2	
	*SCARB1*	1, 2, 3, 5, 7, 8, 10, 13, 14, 16		2, 3, 4, 5, 7, 8, 10, 11, 13, 14, 16		4	
Jersey cross	*BCMO1*	2, 3, 5, 7, 8, 9, 10, 11, 12, 14, 15, 16	3.66 (4.81)	2, 3, 5, 8, 9, 10, 11, 12, 14, 15, 16	3.59 (4.68)	None	1.39 (1.78)
	*BCO2*	1, 2, 3, 4, 5, 6, 7, 8, 9, 10, 11, 12		1, 2, 3, 4, 5, 8, 9, 10, 11		None	
	*SCARB1*	1, 2, 3, 5, 6, 7, 8, 9, 10, 11, 12, 13, 14, 15, 16		1, 3, 5, 6, 7, 8, 10, 11, 12, 13, 14, 15, 16		10	
		14, 15, 16		15, 16			
Gir	*BCMO1*	2, 3, 5, 7, 8, 9, 10, 11, 12, 13, 14, 16	1.86 (3.08)	2, 16	0.82 (1.84)	None	0
	*BCO2*	1, 2, 3, 5, 6, 8, 9, 10, 11, 12		12		None	
	*SCARB1*	1, 2, 3, 4, 5, 6, 7, 8, 10, 11, 12, 13, 14, 15, 16		4, 5, 7, 12, 16		None	
Sahiwal	*BCMO1*	2, 3, 5, 7, 8, 9, 10, 11, 12, 14, 16	2.42 (3.45)	2, 3, 5, 8, 10, 11, 12, 14, 16	2.25 (3.32)	None	0.44 (1.03)
	*BCO2*	1, 2, 4, 5, 6, 8, 9, 10, 11, 12		1, 2, 4, 6, 9, 9, 10, 11, 12		11	
	*SCARB1*	1, 2, 3, 5, 6, 7, 8, 9, 10, 11, 13, 14, 16		2, 3, 5, 8, 9, 10, 11, 13, 16		None	
Tharparkar	*BCMO1*	3, 5, 6, 7, 8, 9, 10, 11, 15	1.69 (2.98)	3, 6, 7, 10, 11, 15	0.67 (1.21)	7	0.32 (0.71)
	*BCO2*	1, 4, 5, 6, 8, 11, 12		4, 8		None	
	*SCARB1*	2, 3, 4, 6, 8, 10, 11, 13, 14, 16		4, 11, 14		None	
Buffalo combined	*BCMO1*	18, 20, 21, 22, 23, 24	1.20 (1.80)	24	0.91 (1.45)	None	0.22 (0.40)
	*BCO2*	14, 15, 16, 17, 18		14, 15, 16		None	
	*SCARB1*	17, 18, 19, 20, 21, 22, 23, 24, 25		19, 20, 21		19	
Jaffarbadi	*BCMO1*	18, 19, 20, 21, 22, 24	4.01 (3.10)	18, 19, 20, 21, 22, 24	4.01 (3,10)	18, 20, 21, 22, 24	3.36 (2.51)
	*BCO2*	13, 14, 16, 17, 18		13, 14, 16, 17, 18		13, 16	
	*SCARB1*	17, 18, 19, 20, 21, 22, 23, 24, 25		17, 18, 19, 20, 21, 22, 23, 24, 25		18, 23	
Murrah	*BCMO1*	18, 20, 21, 22, 24	4.73 (1.99)	18, 24	4.46 (1.91)	None	4.01 (1.21)
	*BCO2*	14, 16, 17, 18		16, 17		16	
	*SCARB1*	17, 18, 19, 20, 21, 22, 23, 24, 25		18, 22, 24		None	
				PP > 0.4		PP > 0.5	
Pandharpuri	*BCMO1*	18, 19, 20, 21, 22, 23, 24	10.42 (7.33)	18, 19, 20, 21, 22, 23, 24	10.20 (7.23)	24	6.87 (3.67)
	*BCO2*	13, 14, 15, 16, 17, 18		13, 14, 15, 16, 17, 18		18	
	*SCARB1*	17, 18, 19, 20, 21, 22, 23, 24, 25		18, 19, 20, 21, 22, 23, 24, 25		21, 22, 23, 25	
				PP > 0.4		PP > 0.5	
Surti	*BCMO1*	18, 20, 21, 22, 23, 24	9.43 (5.67)	18, 20, 23, 24	8.332 (4.89)	24	4.22 (2.99)
	*BCO2*	14, 6, 17, 18		16, 17		16	
	*SCARB1*	17, 18, 19, 20, 21, 22, 23, 24, 25		18, 19, 20, 21, 22, 23, 24		19	

1 No SNP with *P*-value ≤0.3 were found for Mehsana; therefore, no SNP were included in these analyses.

2 Includes all SNP with *P*-value ≤0.3.

3 Represents EBV of an animal homozygous for all favorable alleles.

It is important to note that estimates of total gain in the Bayesian analyses tended to be smaller than those in the frequentist approach due to both the generally smaller number of markers found to be significant and due to the expected shrinkage of their effects in the Bayesian analyses (Bhattacharya et al., 2012). Even though there are clear differences in the magnitude of the substitution effects and the number of significant markers for each breed depending on the analysis method used, the results of our analyses serve as confirmation of the possible applicability of genetic selection for the improvement of nutritional value of milk in regard to BC content and demonstrate that there is value in further investigating the genetic potential of cattle and buffalo breeds for its production.

It is also very important to note that the expression of a phenotype is dependent on the interaction between environment and genotype and most of the animals sampled for this project were under harsh environmental conditions. India is a developing country with rural areas that are often poor, and the animals sampled were under varied and generally suboptimal management and nutritional conditions. Even though a fixed effect for herd was included in our statistical models to account for the different herd conditions found throughout the samples, the generalized less than optimal nutritional, environmental, and management conditions that these animals were kept under might have had an overall negative effect that prevented or decreased the full expression of the phenotypes associated with the concentration of BC in milk. Therefore, improving the aforementioned conditions should go hand in hand with the selection program to successfully and significantly increase the concentration of BC concentration in milk in the cattle and buffalo Indian population.

## CONCLUSIONS

The custom panel designed for genes related to BC production shows applicability in genotyping of cattle and buffalo in India. Among the genotyped SNP, some were significantly associated in several cattle and buffalo breeds, providing markers that may be useful to develop genetic selection strategies that can increase BC content in milk of those populations and could be tested in other developing countries. Moreover, the recommendation of selection of significant specific alleles at the gene markers may provide the direction to effectively increase the BC content in milk in the Indian cattle and buffalo populations. Additional analyses will be required to evaluate a haplotype-based selection for SNP in high LD. Moreover, future genome-wide association studies may reveal additional genes associated with BC or vitamin A in cattle and buffalo. The possible discovery of new candidate genes involved in the BC production would help increase the number of informative SNP in this panel.
